# Colicin Z, a structurally and functionally novel colicin type that selectively kills enteroinvasive *Escherichia coli* and *Shigella* strains

**DOI:** 10.1038/s41598-019-47488-8

**Published:** 2019-07-31

**Authors:** Lenka Micenková, Juraj Bosák, Jiri Kucera, Matěj Hrala, Tereza Dolejšová, Ondrej Šedo, Dirk Linke, Radovan Fišer, David Šmajs

**Affiliations:** 1 Research Centre for Toxic Compounds in the Environment, Faculty of Science, Masaryk University, Kamenice 5, 625 00 Brno, Czech Republic; 2 Department of Biology, Faculty of Medicine, Masaryk University, Kamenice 5, Building A6, 625 00 Brno, Czech Republic; 3 Department of Biochemistry, Faculty of Science, Masaryk University, Kamenice 5, Building A5, 625 00 Brno, Czech Republic; 4 Department of Genetics and Microbiology, Faculty of Science, Charles University, Viničná 5, 128 44 Prague 2, Czech Republic; 5 Central European Institute of Technology, Masaryk University, Kamenice 5, 625 00 Brno, Czech Republic; 6 Department of Biosciences, University of Oslo, P.O. Box 1066, Blindern, 0316 Oslo Norway

**Keywords:** Microbial ecology, Bacterial genes

## Abstract

Colicin production in *Escherichia coli* (*E*. *coli*) strains represents an important trait with regard to microbial survival and competition in the complex intestinal environment. A novel colicin type, colicin Z (26.3 kDa), was described as a product of an original producer, extraintestinal *E*. *coli* B1356 strain, isolated from the anorectal abscess of a 17 years-old man. The 4,007 bp plasmid (pColZ) was completely sequenced and colicin Z activity (*cza*) and colicin Z immunity (*czi*) genes were identified. The *cza* and *czi* genes are transcribed in opposite directions and encode for 237 and 151 amino acid-long proteins, respectively. Colicin Z shows a narrow inhibitory spectrum, being active only against enteroinvasive *E*. *coli* (EIEC) and *Shigella* strains via CjrC receptor recognition and CjrB- and ExbB-, ExbD-mediated colicin translocation. All tested EIEC and *Shigella* strains isolated between the years 1958–2010 were sensitive to colicin Z. The lethal effect of colicin Z was found to be directed against cell wall peptidoglycan (PG) resulting in PG degradation, as revealed by experiments with Remazol Brilliant Blue-stained purified peptidoglycans and with MALDI-TOF MS analyses of treated PG. Colicin Z represents a new class of colicins that is structurally and functionally distinct from previously studied colicin types.

## Introduction

Both commensal and pathogenic *Escherichia coli* strains encode systems for competition among bacterial strains/species allowing survival in the complex intestinal environment. Competition is often driven by the production of bacteriocins, i.e., antibacterial proteins and peptides that selectively kill closely related species^1^. *E*. *coli* strains have been shown to produce two different bacteriocin types including colicins and microcins^2–5^. Microcins are low molecular weight oligopeptides (<12 kDa) compared to colicins which are proteins with molecular weight between 10 and 92 kDa. Microcins can be chromosomally or plasmid encoded, whereas all colicin types are plasmid encoded^3–7^.

From an ecological perspective, bacteriocin production appears to facilitate the invasion of a particular bacterial strain into an established microbial community. It may also mediate defense against invasion of other strains into the occupied niche^8–10^. Moreover, bacteriocinogeny has been shown to be an important feature of probiotic *E*. *coli* strains, typically producing, at least, one bacteriocin type^11^. In addition, an association between the production of several bacteriocin types and the presence of bacterial virulence determinants has been previously described, suggesting that bacteriocins play a role in the colonization capacity of *E*. *coli* strains^12–17^.

Colicins are the most extensively studied bacteriocins produced by Gram-negative bacteria and to date, the production of 25 different colicin types have been clearly demonstrated in *E*. *coli* strains^3,4,7^ with the newest type (type R) having been described in 2014^18^. Until now, all well-characterized colicins were plasmid-encoded domain-containing proteins, except for the plasmid-encoded polypeptide colicin J_S_, where no domains have been identified^7^. Known colicin types differ in a number of characteristics including mechanism of their release from the producer cell, types of translocation systems used to traverse the cell envelope, mechanism of killing action, receptor specificity, as well as several others^3,4^.

In this study, a novel colicin, type Z, isolated from the extraintestinal *E*. *coli* strain B1356 (pColZ) strain is described in detail, including a characterization of the original producer strain, the complete colicin Z plasmid sequence (pColZ), and identification of the genes responsible for colicin activity (*cza*) and immunity (*czi*). Prevalence of the colicin Z activity gene among *E*. *coli* strains of different origins and the colicin Z activity spectrum were tested. In addition, the colicin Z receptor, translocation system, and mode of action were determined.

## Results

### Identification of a colicin Z producer

The original colicin Z producer *E*. *coli* B1356 (Table 1) was identified as extraintestinal pathogenic *E*. *coli* belonging to phylogenetic group D, which contained 4 out of the 20 tested virulence determinants (*fimA*, *iucC*, *aer*, and *pap*). In the *E*. *coli* B1356 strain, none of the tested bacteriocin determinants were detected. From the set of standard indicator strains, the *E*. *coli* B1356 strain inhibited only the growth of *S*. *sonnei* 17. Based on these results we suggested that this was a novel colicin type, encoded by *E*. *coli* B1356 strain, which we named colicin Z.

**Table 1:** Bacterial strains and plasmids used in this study.

Bacterial strains or plasmids	Genotype and/or phenotype	Source
**Bacterial strains**		
*E*. *coli* B1356	Original producer of new colicin type Z; extraintestinal pathogenic *E*. *coli* strain isolated from the anorectal abscess of a 17-years-old man	This laboratory
*E*. *coli* DH10B	F^–^ *endA1 deoR*^+^ *recA1 galE15 galK16 nupG rpsL* Δ*(lac)X74* φ80*lacZΔM15 araD139* Δ*(ara*,*leu)7697 mcrA* Δ*(mrr-hsdRMS-mcrBC)* Str^R^ λ^−^	Thermo Fisher Scientific
*E*. *coli* (*E*. *cloni*^®^ 10G)	F^−^ *mcr*A Δ(*mrr*-*hsd*RMS-*mcr*BC) *end*A1 *rec*A1 Φ80d*lac*ZΔM15 Δ*lac*X74 *ara*D139 Δ(*ara*,*leu*)7697 *gal*U *gal*K *rps*L (Str^R^) *nup*G λ- *ton*A	Lucigen
*E*. *coli* TOP10F′	F′{lacIq Tn10 (TetR)} *mcrA* Δ(*mrr*-*hsd*RMS-*mcr*BC) Φ80lacZΔM15 Δ*lac*X74 *rec*A1 *ara*D139 Δ(*ara*-*leu*)7697 *gal*U *gal*K *rps*L *end*A1 *nup*G	Thermo Fisher Scientific
*E*. *coli* A592 Δ*tol*A	Δ*tol*A; containing pBAD/HisB with *cjr*BC genes	This laboratory
*E*. *coli* A593 Δ*tol*B	Δ*tol*B; containing pBAD/HisB with *cjr*BC genes	This laboratory
*E*. *coli* CGSC 7437 Δ*tol*C	Δ*tol*C; containing pBAD/HisB with *cjr*BC genes	This laboratory
*E*. *coli* TPS13 Δ*tol*Q	Δ*tol*Q; containing pBAD/HisB with *cjr*BC genes	This laboratory
*E*. *coli* TPS300 Δ*tol*R	Δ*tol*R; containing pBAD/HisB with *cjr*BC genes	This laboratory
*E*. *coli* CGSC 5415 Δ*ton*B	Δ*ton*B; containing pBAD/HisB with *cjr*BC genes	This laboratory
*E*. *coli* BR158 Δ*ton*B	Δ*ton*B; containing pBAD/HisB with *cjr*BC genes	This laboratory
*E*. *coli* BW25113-K12 WT	Control strain - wild type	Keio collection
*E*. *coli* CGSC 5417 Δ*exb*B	Δ*exb*B; containing pBAD/HisB with *cjr*BC genes	This laboratory
*E*. *coli* JW2973 Δ*exb*D	Δ*exb*D; containing pBAD/HisB with *cjr*BC genes	This laboratory
**Plasmids**		
pBAD/HisB	Cloning vector; *ara* promoter transcription-translation system with N-terminal His tag	Thermo Fisher Scientific
Colicin Z activity and immunity		
pColZ46	pBAD/HisB with *cza* (colicin Z activity) gene	This laboratory
pColZ99	pBAD/HisB with *czi* (colicin Z immunity) gene	This laboratory
Colicin Z receptor		
pColZ91	pBAD/HisB with *cjr*A gene	This laboratory
pColZ93	pBAD/HisB with *cjr*B gene	This laboratory
pColZ95	pBAD/HisB with *cjr*C gene	This laboratory
pColZ97	pBAD/HisB with *cjr*AB genes	This laboratory
pColZ123	pBAD/HisB with *cjr*BC genes	This laboratory

### Characterization of pColZ and identification of the colicin activity and immunity genes

**Figure 1:**
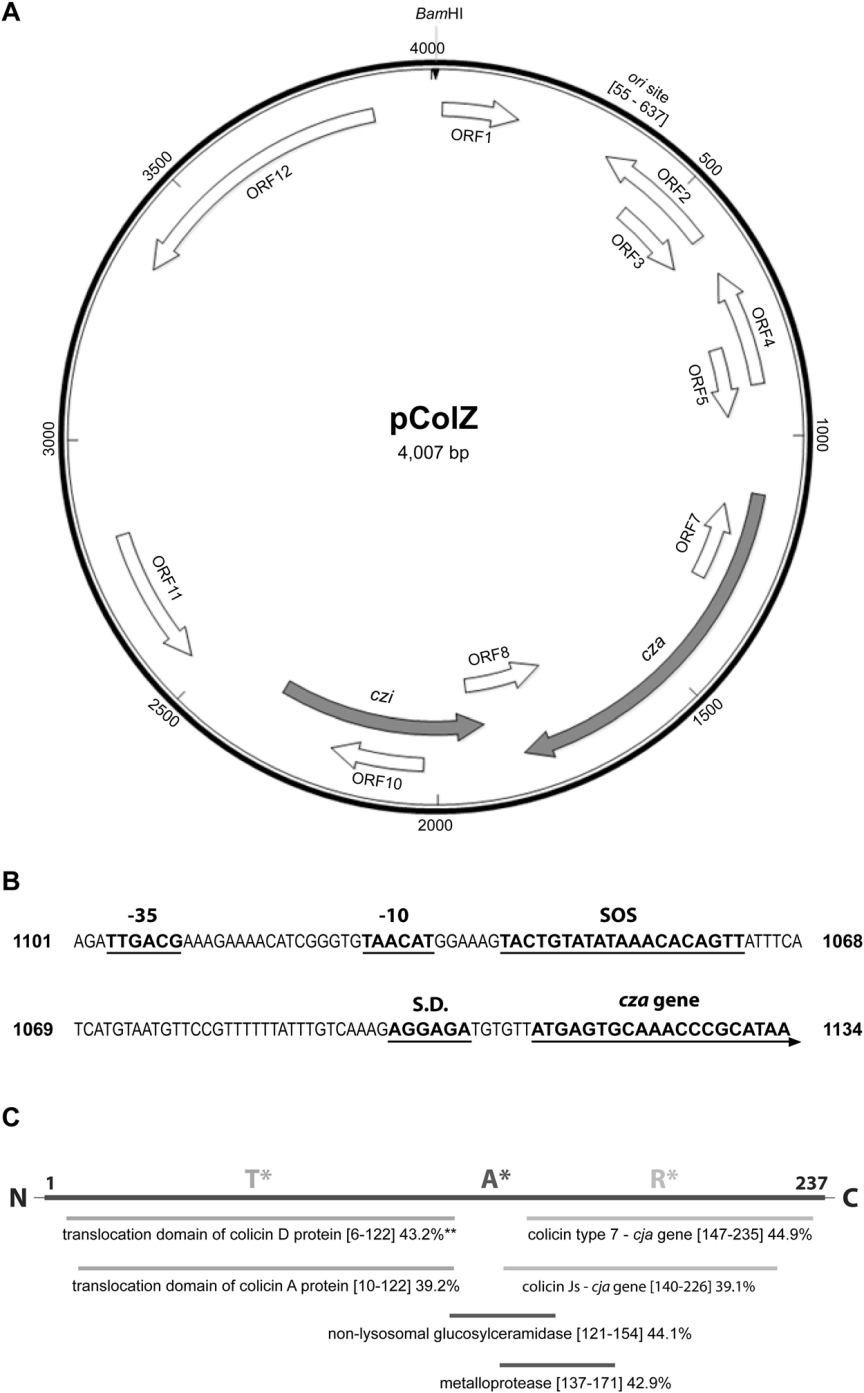
(**A**) A schematic map of plasmid ColZ (4,007 bp). Unique *Bam*HI restriction site was used as a reference point in the pColZ genetic map. The localization and polarity of predicted genes and the position of the putative origin of replication (*ori*) are indicated. The colicin Z activity (*cza*) and immunity genes (*czi*) are shown as gray arrows. (**B**) Putative promoter regions (−10 and −35), SOS box, and ribosome binding site sequences (S.D.) are indicated. Numbers correspond to positions in pColZ. (**C**) A domain organization of colicin Z. The N-terminal part of colicin Z protein sequence showed similarity to the colicins A and D translocation domain. Homology to non-lysosomal glucosylceramidase and metalloprotease was seen in the central part of colicin Z and the T-terminal part showed homology to colicin J_S_. Functional domains of colicin J_S_ have not been previously described, but colicins Z and J_S_ use the same receptor molecule, i.e., protein CjrC. *predicted translocation, activity, and receptor binding domains **sequence identity (%).

The colZ plasmid consists of 4,007 base pairs with a GC content of 43.2%. The complete nucleotide sequence of plasmid ColZ was deposited in the GenBank under accession number MK5999282. pColZ has a unique *Bam*HI restriction site, which was used as a reference point in the presentation of the pColZ genetic map (Fig. 1A). pColZ comprises 12 predicted open reading frames (ORFs) encoding polypeptides longer than 50 amino acids (Table 2). The putative origin of plasmid replication was identified between positions 55 bp to 637 bp on pColZ and is similar (35.6%) to the *ori* site of *S*. *flexneri* plasmid pSF301-1 (GenBank acc. no. JF813186). A primosome assembly site for pColZ was identified between positions 82 and 147, and this 66 bp sequence was 98.5% identical to the phiX174 primosome assembly site identified in plasmid pHUSEC. 2011-3 (Gen Bank acc. no. HE610902). A 156 bp-long ORF1 with 56.4% similarity to the plasmid replication gene and also a 153 bp-long ORF5 with low identity 45.7% to the *mobB* gene were identified on pColZ (Table 2).

**Table 2:** Characterization of pColZ predicted open reading frames (ORFs) encoding polypeptides longer than 50 amino acids.

ORF	Strand	Frame	Start	Stop	Lenght (aa)	Protein similarity	UniProt	Organism	No. of aa aligned	Identity (%)
1	+	1	13	168	51	Probable plasmid replication initiation protein	A0A2X6I7G5	*E*. *coli*	39	56.4
2	−	1	590	348	80	Rep protein	A0A152X0V5	*E*. *coli*	76	93.4
3	+	3	441	611	56	Plasmid replication protein	A0A2Y0VLC8	*E*. *coli*	50	96.0
4	-	3	897	667	76	Plasmid mobilization protein MobB	A0A2Y8HSM0	*E*. *coli*	45	36.5
5	+	2	809	961	50	MobB	M1L7H5	*Edwardsiella ictaluri*	35	45.7
6 (*cza*)	+	1	1114	1827	237	Colicin-A protein	U2N7R4	*Serratia fonticola*	118	39.2
						Colicin type 7 (Cja)	A0A0T9U2E6	*Yersinia enterocolitica*	89	44.9
7	−	1	1310	1146	54	Colicin-A protein	U2N7R4	*Serratia fonticola*	54	32.8
8	−	1	1928	1734	64	Uncharacterized protein	U5TRH2	*Serratia marcescens*	30	76.7
9 (*czi*)	−	3	2346	1891	151	Uncharacterized protein	A0A1X1EJN9	*Pantoea cypripedii*	145	33.8
10	+	2	2027	2209	60	Uncharacterized protein	A0A1X0VY47	*Rouxiella silvae*	56	35.7
11	−	3	2811	2539	90	Helix-turn-helix domain-containing protein	A0A2T1MMQ2	*E*. *coli*	74	91.9
12	−	3	3885	3346	179	Firmicute plasmid replication protein	E9TMC2	*E*. *coli*	175	98.3

Using a BLAST search for amino acid sequence similarities in the UniProt database (Table 2), an ORF6 (714 bp) at plasmid positions 1114–1827 was identified as the putative colicin Z activity gene (*cza*) based on its low similarity (44.9%) to colicin type 7 (colicin J_S_) from *Yersinia enterocolitica* (GenBank acc. no. A0A0T9U2E6) and (39.2%) to colicin A from *Serratia fonticola* AU-AP2C (GenBank acc. no. U2N7R4). The ORF6 gene was cloned (resulting in pColZ46) (Table 1) and the recombinant strain *E*. *cloni* pColZ46 inhibited the growth of the indicator strain *S*. *sonnei* 17 indicating the correct identification of *cza*. A final concentration of 0.02% arabinose was used for induction of *E*. *cloni* pColZ46 and for visualization of inhibition zone on indicator strain. Putative promoter regions (−10 and −35), SOS box, and ribosome binding site sequences (S.D.) are indicated in the Fig. 1B. The promoter region upstream of *cza* gene contains a highly conserved −35 region (TTGACG), and a less-conserved −10 region (TAACAT). Similar promoter regions were described upstream of colicin activity gene on pColE1 and pColJ_S_^7,19^. While majority (over 75%) of enteric bacteriocin promoters contain two overlapping SOS boxes, the pColZ have a single SOS box similar to promoters of klebicins C and D^20^. The inductibility of colicin Z synthesis by the SOS response was measured after mitomycin C treatment (0.5 μg ml^−1^). Highest colicin Z dilution that resulted in a clear zone of growth inhibition and the last dilution that resulted in the turbid zones on the lawn of sensitive indicator strain *E*. *coli* O164 was measured. Induction of colicin Z in the culture supernatants led to increase in colicin activity of 1 to 2 orders of magnitude in both, the original producer *E*. *coli* B1356 and in the recombinant strain *E*. *coli* pColZ46.

The Colicin Z immunity gene (*czi*, ORF9) was found adjacent to the *cza* gene and is transcribed in the opposite direction compared to *cza*. The *czi* gene showed 34.7% identity to a hypothetical protein from *Serratia rubidaea* (WP_054306871.1). A recombinant plasmid pColZ99, containing *czi*, was transformed to *E*. *coli* O164 (a strain susceptible to colicin Z) and induction with 0.02% arabinose resulted in resistance to colicin Z. The colicin Z immunity protein was predicted as a cytoplasmic membrane protein with four transmembrane segments, similar to colicin A immunity protein^21^. No signal peptide sequence was identified in the colicin Z immunity protein.

The average GC content of the *cza* and *czi* genes was found to be 39%, while that of remaining plasmid was 45%, suggesting acquisition through horizontal gene transfer.

The deduced amino acid sequence (237 amino acids) of colicin Z (26.3 kDa) was compared with amino acid sequences of 25 previously characterized colicin types. Colicin J_S_, the smallest known colicin type (10.4 kDa) and first identified in *S*. *sonnei* strain^7^, was found as the most closely related colicin type (Supplementary Fig. S1). The Colicin Z immunity protein contains 151 amino acids with a calculated molecular mass 17.7 kDa, and showed no similarity to previously described colicin immunity proteins. While N-terminal part of colicin Z protein sequence shows similarity to the translocation domains of colicin D and colicin A (Fig. 1C, Supplementary Fig. S2), the central domain between the amino acids 121 and 171 shows homology to a non-lysosomal glucosylceramidase (GenBank acc. no. A0A0A9X393) and a metalloprotease (A0A060LZ15). The C-terminal part of colicin Z protein shows similarity to colicin J_S_ (A0A0T9U2E6).

### Colicin Z inhibitory spectrum

*E*. *coli* B1356 producing colZ was active against only 6.7% (38 from 563) of the tested bacteria and its inhibitory spectrum comprised EIEC O143 and O164 strains, as well as different *Shigella* strains (Table 3, Supplementary Table S1).

**Table 3:** Colicin Z activity spectrum and prevalence of *cza* gene in the sets of *E*. *coli* strains.

Genus/Strain	No. of strains	Sensitivity to colicin Z
E. coli strains	No. osf strains	Prevalence of cza gene (%)
**Colicin Z activity spectrum**		
*Budvicia*	3	no
*Citrobacter*	3	no
*Enterobacter*	1	no
Enterohemorrhagic *E*. *coli*	152	no
Enterotoxigenic *E*. *coli*	200	no
ESBL-producing *E*. *coli* ST131	4	no
commensal *E*. *coli*	46	no
enteroinvasive *E*. *coli*	2	yes (all sensitive)
*E*. *fergusonni*	2	no
*Klebsiella*	1	no
*Kluyvera*	1	no
*Leclercia*	3	no
*Micrococcus*	1	no
*Pragia*	3	no
*Proteus*	1	no
*Sallmonella*	2	no
Shiga-toxigenic *E*. *coli*	101	no
*Shigella*	36	yes (all sensitive)
*Staphylococcus*	1	no
**Prevalence of cza gene in E. coli strains**		
Commensal	95	n.d.**
Uropathogenic	95	n.d.
Extraintestinal pathogenic	95	n.d.
Intestinal pathogenic*	95	n.d.
Veterinary	95	n.d.

*Intestinal *E*. *coli* isolated from patients with diagnosed infectious and parasitic diseases

**N.d. not detected.

### Prevalence of colicin Z gene among *E*. *coli* strains

The *cza* gene was not detected in any of tested *E*. *coli* strains (n = 475) (Table 3, Supplementary Table S2).

### Identification of colicin Z receptor and translocation of colicin Z to a susceptible bacterium

Based on the antimicrobial spectrum of colicin Z, which showed similarity to the colicin J_S_ inhibitory spectrum^7,22^, the *cjr*ABC operon encoding colJ_S_ receptor (GenBank acc. no. AF283288.1) was analyzed (Table 4). Recombinant pBAD/HisB plasmids containing combinations of *cjr* genes were constructed and transformed to *E*. *coli* DH10B cells (Table 4). Acquisition of the *cjr*BC genes resulted in susceptibility to colZ (Table 4). For identification of the colZ translocation system, a set of knockout strains were transformed using a pBAD/HisB vector containing *cjr*BC genes (Table 1) and tested for susceptibility to colZ. An *exb*B and *exb*D mutants were not susceptible to colZ, while *ton*B mutants and *tol*A, -B, -Q, were susceptible. ColZ recognizes CjrC receptor and uses CjrB- and ExbB-, ExbD-mediated translocation.

**Table 4:** Sensitivity of *E*. *coli* DH10B cells carrying different plasmids to colicin Z.

Plasmids	Genes introduced	Colicin Z sensitivity
None	—	resistant
pColZ91	*cjr*A gene	resistant
pColZ93	*cjr*B gene	resistant
pColZ95	*cjr*C gene	resistant
pColZ97	*cjr*AB genes	resistant
pColZ123	*cjr*BC genes	sensitive

### Colicin Z purification and mode of killing action

**Figure 2:**
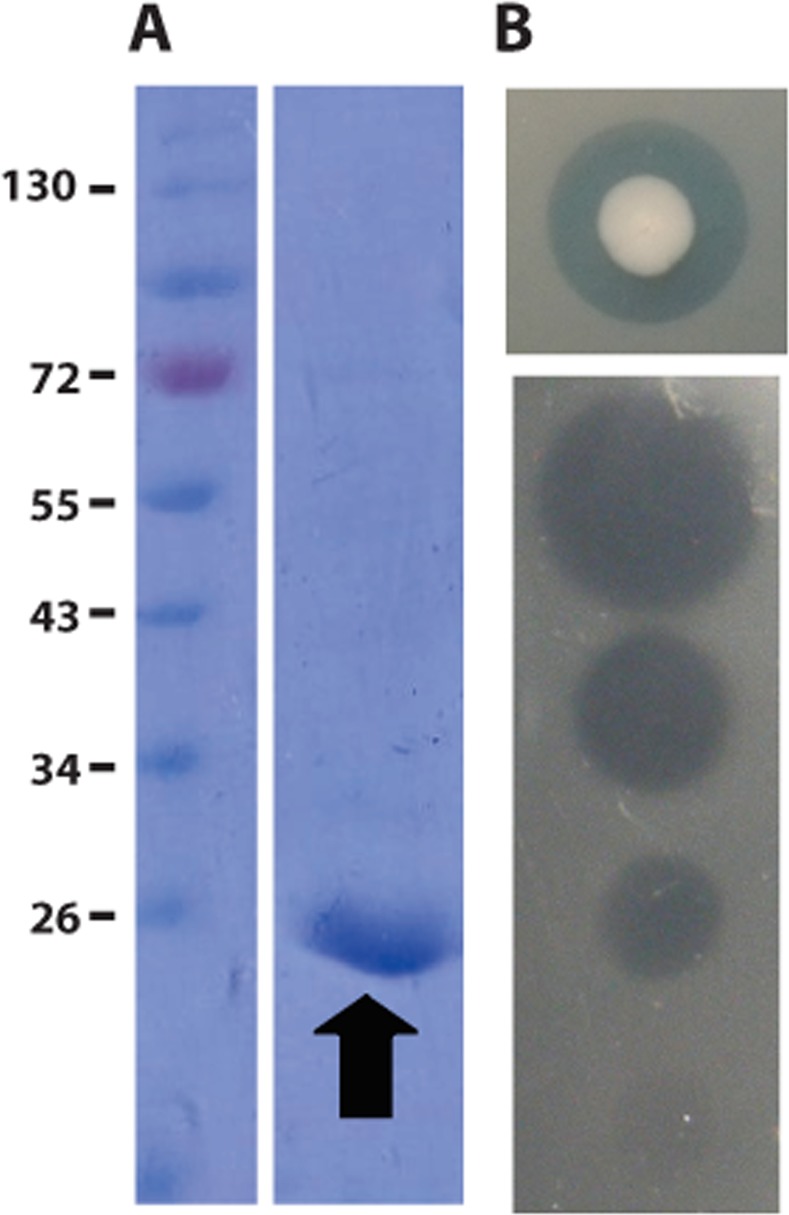
Purification of colicin Z and its biological activity. (**A**) Purification of colicin Z containing an N-terminal histidine tag by using Ni Sepharose 6 Fast Flow column. Lane 1, low-molecular-weight protein standard (PageRuler Prestained Protein Ladder, Fermentas); lane 2, purified colicin Z with an N-terminal histidine tag. Full-length gel is included in a Supplementary Fig. S3. (**B**) Inhibition zone of the original producer of colicin Z (*E*. *coli* B1356), against the *E*. *coli* O164 indicator strain and the antibacterial activity of purified N-terminal His-tagged colicin Z (tested by spotting of 10-fold serial dilutions) on the *E*. *coli* O164 strain. The biological activity of purified colicin Z was 10^4^ arbitrary units.

According to the transcriptional orientation of the colicin Z activity and immunity genes on the ColZ plasmid, the mode of colicin Z action is consistent with either pore-forming or peptidoglycan (PG)-directed activity but not with DNA or RNA nuclease activity^3,4^. *E*. *coli* TOP10F′containing the pColZ46 encoding colicin Z with an N-terminal His tag was constructed and used for colicin Z purification. Following purification, colicin Z (26.3 kDa) activity was 10^4^ arbitrary units per µl (Fig. 2A,B; Supplementary Fig. S3).

Potential pore-forming activity of purified colicin Z protein was tested by conductance measurements on black lipid membranes. Extremely low activity was detected on asolectin membranes and membranes made from *E*. *coli* polar extracts. Moderate pore-forming activity was detected on membranes made from synthetic phospholipid DPhPG (1,2-diphytanoyl-sn-glycero-3-phospho-(1′-rac-glycerol)) (pores of conductance about 2.8 pS). Low single-pore conductance together with weak pore-forming activity was considered inconsistent with pore-formation as the principal mode of action of colZ.

**Figure 3:**
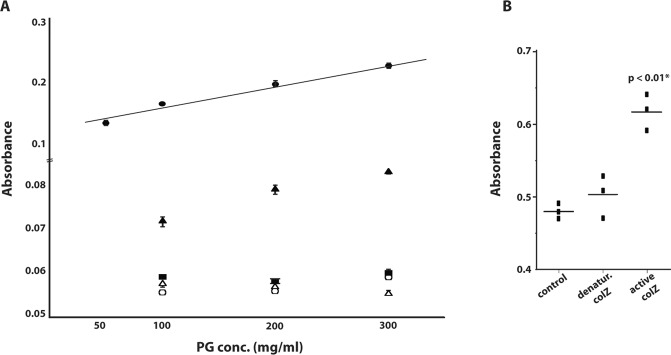
(**A**) Absorbance of dye released from the samples of RBB-stained peptidoglycan incubated (24 h at 37 °C) with active purified  colicin Z,  lysozyme and controls ( PG + distilled water;  PG + purified control *E*. *coli* TOP10F′ harboring only cloning vector pBAD/HisB; and  PG + denatured purified colZ) measured at 595 nm. (**B**) Absorbance of dye released from control samples of RBB-PG and from RBB-PG treated with denatured or active colicin Z. A statistically significant difference was observed between the activity of denatured and active colicin Z.

Remazol Brilliant Blue (RBB)-stained purified peptidoglycan (see Methods) was used to test the activity of purified colZ on PG. The absorbance of dye released from the samples of RBB-PG incubated with active, purified colicin Z and controls including lysozyme, denatured purified colZ, distilled water, and purified lysate of *E*. *coli* TOP10F′ pBAD/HisB (without the *cza* gene) was measured at 595 nm. An average of three independent measurements (each with two technical replicates) is shown in Fig. 3A. An increasing signal was detected with an increased concentration of active colZ and lysozyme but not with negative controls. Compared to negative control samples, including denatured colZ sample, active colZ showed higher activity (t-test; p < 0.01) (Fig. 3B).

**Figure 4:**
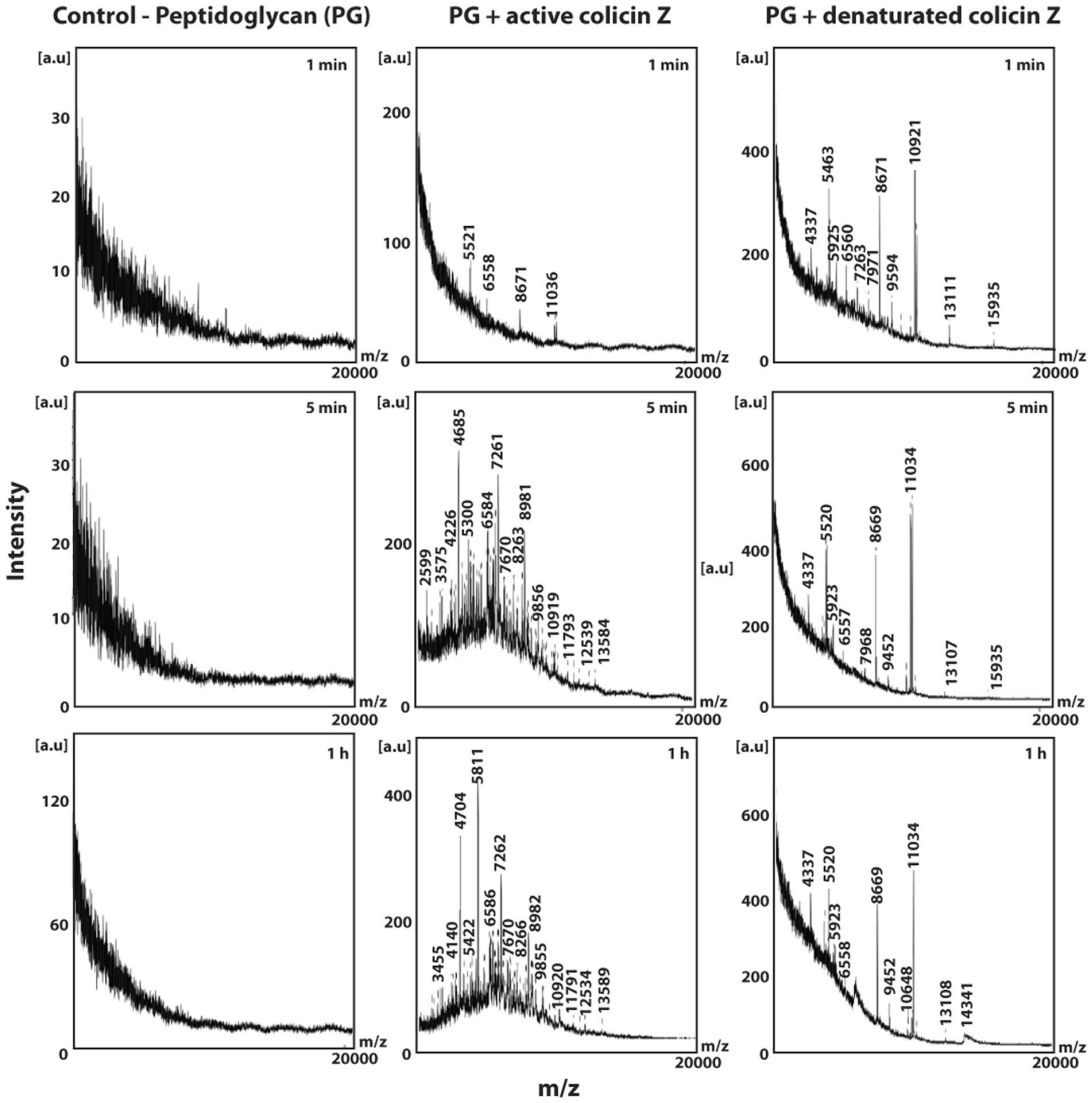
MALDI-TOF MS analysis of peptidoglycan cleavage products following incubation with active and denatured colicin Z. In the MALDI-TOF MS profiles of control PG and PG incubated with denatured colicin Z (0.1 mg/ml), no difference was detected during the time-points tested (1 min, 5 min, and 1 hour). In the samples containing PG incubated with active colicin Z (0.1 mg/ml), a variety of new peaks in the range from 2500 to 14000 m/z were detected after 5 min of incubation. These peaks are consistent with the different products of peptidoglycan degradation.

MALDI-TOF MS analysis was used to detect the cleavage products of peptidoglycan incubated with active or denatured colicin Z (Fig. 4). In the MALDI-TOF MS profiles of water-treated PG and PG incubated with denatured purified colicin Z (0.1 mg/ml), no difference was detected during the assessed time-points (i.e., 1 min, 5 min, and 1 hour). In the samples containing PG and the active colicin Z preparation (0.1 mg/ml), a variety of new peaks in the range from 2500 to 14000 *m*/*z* was detected following 5 min of incubation.

## Discussion

In this work we have identified a novel 26.3 kDa colicin, designated as colicin Z, produced by *E*. *coli* strain B1356, with a human origin and active against both EIEC and *Shigella* strains. Altogether, twenty-five colicin types^3,4,18^ have been previously characterized and all of them showed a protein domain structure. The only exception was colicin J_S_, which was found to be considerably smaller than other colicin types with no protein domains identified so far^7^. The modular structure of colicins appears to allow frequent combinations of the corresponding domains to create new toxic functions in a competitive environment^23–25^. The N-terminal domain of colicin Z showed a low similarity (~40%) to the translocation domain of colicins D and A, and to protein translocase which is in agreement with the localization of translocation domains at the N-termini of most colicin types. Colicin A is Tol-dependent colicin type and colicin D is TonB dependent^4^ but comparison between translocation domains of colA and colD revealed similar identity (~40%) like comparison of predicted colZ T-domain with colA or colD T-domains. However, the colicin Z protein sequence at the C-terminus shows similarity to colicin J_S_. Information about colicin J_S_ domain organization is not known but given the fact that the activity spectra of colicins J_S_ and colicin Z were both limited to EIEC and *Shigella* strains, the C-terminal domain is likely responsible for receptor binding to the CjrC receptor molecule. Following similarity of N-terminal domain of colicin Z to translocation proteins and to translocation domains of colicins A and D, appears unlikely that C-terminal sequence of colicin Z is responsible for the recruitment of the CjrB and ExbBD proteins and for the translocation of colicin Z. The central protein domain of colicin Z shows similarities to enzymes that degrade glycolipids and polypeptides but no similarity to other colicin types suggesting that it is the central part of colicin Z molecule that exerts its (novel) lethal effect. Although not fully experimentally verified, the domain organization of colicin Z appears to be in contrast to previously described colicin types where the central part of the protein is responsible for receptor binding and the C-terminal for the killing activity^4^. Several S-type pyocin types have their receptor domain at the N-terminus of their molecules^26,27^ indicating that the positions of individual domains in the antibacterial molecules are not strictly conserved and that colicins could have this unusual molecular organization.

Among the colicin types described to date^3,4,18,28^, only colicin J_S_^7^ and colicin Z selectively kill EIEC and *Shigella* strains. Enteroinvasive *E*. *coli* represent the etiological agents of bacillary dysentery and cause proctocolitis *via* a nontoxigenic mechanism similar to *Shigella* strains. In fact, both EIEC and *Shigella* strains have been shown to be highly related^29^. The selective activity of colicin Z on EIEC and *Shigella* strains is a result of the requirement of *cjr*BC genes (harbored by both EIEC and *Shigella* strains) for receptor binding and colicin translocation. In addition to the *cjr*BC genes, colicin Z activity was found to be dependent on ExbB and ExbD. Colicin Z is, similarly as colicin J_S_, dependent on ExbB. While colicin Z is also dependent on ExbD protein, Δ*exb*D strains were not previously tested with colicin J_S_^22^. CjrB shows similarities to TonB from Gram-negative bacteria and appears to be an EIEC-specific TonB homolog^26^. CjrC is a homolog of the outer membrane receptors responsible for colicin J_S_ uptake as well as for possible siderophore or heme uptake by Gram-negative bacteria^18^. ExbB and ExbD are transmembrane proteins of the inner membrane that energize the TonB protein in Gram-negative bacteria^30^. In known TonB-dependent colicin types (B, Ia, Ib, D, 5, 10, and M), a pentapeptide sequence motif, the TonB-box, was identified near the N-terminus. In colicin Z, similarly to colicin J_S_^7^, the TonB box sequence motif was not found and the absence of the TonB box likely results in the *ton*B-independent translocation of colicin Z and J_S_ mediated by CjrB. Interestingly, while the primary structure of colicin J_S_ and colicin Z are quite different, both molecules are directed against EIEC and *Shigella* strains suggesting that there is an evolutionary need for the existence of protein molecules that can kill EIEC and *Shigella* strains. Binding of different colicin types to identical receptor molecules is common and includes BtuB, FepA, Fiu, Cir, and Tsx outer membrane proteins^3^.

Colicin Z is encoded on a pColZ plasmid that is slightly over 4 kb (4,007 bp). pColZ thus represents the smallest known colicinogenic plasmid; the sizes of previously described colicinogenic plasmids ranged from 5.2 kb to over 75 kb^3,4^. About 1 kb of pColZ was homologous to plasmid pSF301-1 identified in (*S*. *flexneri*) and to plasmid pEC732_5 (*E*. *coli*), mainly in regions responsible for plasmid replication, suggesting a common origin of these plasmids. In the remaining part of pColZ, no sequence similarity to previously described plasmids was found. These findings, together with detected differences in the GC content of colicin Z operon and the remaining part of the colicin Z plasmid, suggesting that the pColZ evolved, through DNA recombination from the different sources, relatively recently. No virulence genes were identified on pColZ. Therefore we assume that the main role of this plasmid is colicin Z production.

As revealed through the screening of human commensal and pathogenic as well as veterinary *E*. *coli* strains, colicin Z appears to be an extremely rare colicin type among these strains. Previous studies have shown differences in the prevalence of diverse colicin types among the sets of commensal and pathogenic *E*. *coli* strains^15–17,31^ with frequencies ranging from 0.1 to 5% for rare colicin types (e.g., colicin N, S4, U, Y, J_S_) to more than 10% for frequently detected colicin types (e.g., Ia, E1, and M)^14–17,31^. The rare occurrence of colicin Z producers appears to be associated with the common susceptibility of EIEC and different *Shigella* strains to colicin Z. All strains in the set of tested *Shigella*/EIEC strains (n = 38) isolated between the years 1958–2010 were susceptible to colicin Z. A similar situation was found among strains susceptible to colicin F_Y_, where all tested strains of *Yersinia enterocolitica* were found susceptible to colicin F_Y_ produced by *Y*. *frederiksenii*^28^, likely as a result of the sporadic co-occurrence of both producer and susceptible species^32^. The extremely rare occurrence of colicin Z producers among tested strains suggests that either colicin Z producers are primarily present in other strains than those tested or it represents sporadic producer strains. In any of these cases, the absence of colicin Z producers may explain the complete susceptibility of the tested EIEC and *Shigella* strains to colicin Z. In contrast, for several other colicin types, it is estimated that 75% of *E*. *coli* strains isolated from different sources are resistant to one or more bacteriocin types and that the resistance to bacteriocins is a successful strategy in antimicrobial competition^9,31,33,34^.

The colicin Z immunity gene is located downstream from the *cza* gene with an opposite transcription polarity. Immunity genes with opposite transcription polarity are typical for colicin types acting on the plasma membrane of susceptible bacteria or for colicin M, which inhibits peptidoglycan synthesis^4,35^. In the killing activity of colicin Z tested with artificial membranes, colicin Z showed almost no pore-forming activity on asolectin membranes. In general, pore-forming colicins are usually very active on asolectin membranes, e.g., colicin A exerts pore-forming activity in the pM concentration range^36^. Another striking difference was the conductance of individual pores. Colicin A and B are known to form very low-conductance pores of 16 and 21 pS^37^, respectively, which is still about 6–8 times greater than the conductance of colicin Z pores (2.8 pS). Therefore, our data were inconsistent with the pore-forming activity of colicin Z and the detected pores with low conductance likely reflect some minor contamination of the purified colicin Z preparation.

In the central colicin Z domain, potentially responsible for the lethal effect of colicin Z, a similarity to non-lysosomal glucosylceramidases, metalloproteases, DUF500, and SH3 domain-containing proteins was found. The bacterial Src homology 3 (SH3) domain was reported to bind to the bacterial cell wall^38–40^, and the ‘SH3-like’ domain represents a non-catalytic domain commonly found in bacterial peptidoglycan hydrolases that may interact with carbohydrates and/or poly-proline stretches^41^. These similarities, together with the transcriptional orientation of the *czi* gene points to the possibility of colicin Z activity on peptidoglycan. These predictions were further supported by the morphological changes of an indicator bacteria in the transient parts of inhibition zones^42^, where colicin Z effects resembled the effect of colicin M (data not shown). As shown by peptidoglycan dye-release assays and with MALDI-TOF MS analyses, colicin Z is involved in the degradation of peptidoglycan. Compared to lysozyme (Fig. 3A), the activity of colicin Z was considerably lower and represented only about 15% of lysozyme activity. However, colicins undergo protein unfolding and refolding during receptor binding and translocation^4^, therefore, soluble colicin Z may have limited activity while lysozyme shows optimum activity in soluble form.

Colicin Z is a novel colicin type that is partially similar to previously described colicin J_S_, having a narrow inhibitory spectrum and being active only against EIEC and *Shigella* strains. Colicin Z thus belongs to the group of colicin types (E1, F_Y_, U, Y, and J_S_) that have been shown to specifically inhibit pathogenic bacteria under *in vitro* or *in vivo* conditions^22,28,32,43–45^. At the same time, there appears to be wide susceptibility of EIEC and *Shigella* strains to colicin Z. These properties of colicin Z suggest a possible future use in therapeutic applications for intestinal shigellosis.

## Methods

### Bacterial strains

The colicin Z producer strain *E*. *coli* B1356 was identified during the screening for bacteriocin production in sets of *E*. *coli* (n = 2160) isolated in Brno, Czech Republic^13,15–17^, unpublished results] (Table 1).

The standard colicin indicator strains *E*. *coli* K12-Row, C6 (ϕ), 5 K, P400, S40, and *Shigella sonnei* 17, capable of detecting all known colicin types, were used^13,15^ in this screening test for bacteriocin producers. In addition, sets of different pathogenic and non-pathogenic strains belonging to different genera (n = 563) were used to identify the inhibitory spectrum of the novel colicin Z (Supplementary Table S1).

The standard bacterial strains were used for cloning, transformation, and protein recombinant expression and purification experiments (Table 1).

A set of knockout strains with gene deletions from the Tol and Ton systems were used^25,46,47^ [Keio collection, K. Hantke collection] (Table 1).

Prevalence of the colicin Z activity gene (*cza*) was screened using sets of human and veterinary *E*. *coli* (n = 475) (Supplementary Table S2).

### Characterization of colicin Z original producer

A total of 20 virulence genes (*α-hly*, *afaI*, *aer*, *cnf1*, *sfa*, *pap*, pCVD432, *ial*, *lt*, *st*, *bfpA*, *eaeA*, *ipaH*, *iucC*, *fimA*, pks island, *cdt*, *ehly*, *stx1*, and *stx2*) and genetic determinants allowing classification into phylogenetic groups (A, B1, B2, and D) were analyzed in *E*. *coli* B1356. PCR screening for bacteriocin determinants encoding 24 colicin types (A, B, D, E1-9, Ia, Ib, Js, K, L, M, N, S4, U, Y, F_Y_, and 5/10) and 7 microcin types (mH47, mM, mB17, mC7, mJ25, mL, and mV) was performed. Primer sequences and PCR protocols were previously described^15,17,48,49^.

### Colicin Z activity spectrum

Identification of the colicin Z inhibitory spectrum was performed as described previously^13^. Briefly, agar plates were inoculated with a stab from a culture of *E*. *coli* B1356 and cultivated for 48 hours (37 °C). The bacteria were then killed using chloroform vapors (30 min) and the plates were then overlaid with a thin layer of 0.7%, agar containing 10^7^ cells ml^−1^ of an indicator strain (overall; n = 563) (Supplementary Table S1) and incubated at 37 °C overnight.

### Recombinant DNA methods

For identification of the pColZ plasmid, transposon mutagenesis with a Tn5 transposon (EZ-Tn5™ < KAN-2 > Insertion Kit; Epicentre Biotechnologies, Madison, WI, USA) was used. Briefly, total plasmid DNA from the original producer of colicin Z was isolated using a QIAGEN Plasmid Midi Kit (Qiagen, Germany). This DNA was used in an *in vitro* transposon insertion reaction and transformed into *E*. *coli* DH10B. Plasmid DNA from 24 recombinant colonies (pColZ1-24) was isolated and the DNA in the vicinity of the inserted Tn*5* transposon was sequenced (Eurofins Scientific, Brussels, Belgium) with Tn5 transposon sequencing primers (Supplementary Table S3). Next, sequencing primers were designed (Table S3) and plasmid DNA of recombinant strain ColZ1 (which was able to inhibit EIEC O164 indicator strain was used for sequencing of pColZ plasmid).

A non-enzymatic *in vivo* cloning strategy was used for recombinational cloning to *E*. *cloni*® 10 G (Lucigen, Middleton, WI, USA). For *in vivo* recombination, a digested pBAD/HisB vector (Thermo Fisher Scientific) was prepared and incubated with purified PCR product with 25 nt-long overlaps (Supplementary Table S3) that were complementary to the vector DNA.

### Prevalence of the colicin Z activity gene (*cza*) among *E*. *coli* strains of different origins

Overall, 475 various *E*. *coli* (Supplementary Table S2) were tested using colony PCR specific for *cza* gene. The PCR detection protocol was as follows: 94 °C (5 minutes); 94 °C (30 seconds), 60 °C (30 seconds), 72 °C (1 minute), 30 cycles; 72 °C (7 minutes). The *cza*-F: 5′-ATGAGTGCAAACCCGCATA-3′and *cza*-R: 5′-TTACTTAGGAAAATCGAAAGTAA-3′ primer pair was used.

### Identification of colicin Z receptor

Based on the antimicrobial spectrum of colicin Z which showed similarity to the colicin J_S_ inhibitory spectrum^7,22^, the role of three genes in the *cjr* operon (responsible for colJ_S_ susceptibility) was analyzed. Combinations of *cjr*ABC genes were cloned into a pBAD/HisB vector (Table 1 and S3) and transformed to *E*. *cloni*® 10 G. Clones were verified by sequencing. Next, agar plates were inoculated with a stab of the bacterial culture of *E*. *coli* B1356 producer and susceptibility of the different recombinant clones to colZ was tested.

### Identification of the colicin Z translocation mechanism

To investigate whether colicin Z is translocated by the Tol or Ton translocation system, different knockout strains were transformed with the recombinant plasmid encoding the colZ receptor - pColZ123 (Table 1). Based on the susceptibility of recombinant clones to colicin Z, the translocation system for colZ was identified.

### Colicin Z purification

Recombinant *E*. *coli* TOP10F′ strain containing pColZ46 encoding the colicin Z activity gene fused to an N-terminal His tag was constructed and used for purification of the colZ protein. The recombinant strain was cultivated in 20 liters of TY medium (37 °C; 100 rpm) until OD_600_ reached 0.6, then colicin expression was induced by L-( + )-arabinose (0.2 g/l, Sigma-Aldrich) and cultivated for a further 4 hours (37 °C; 100 rpm). Cells were harvested and frozen at −80 °C. A lysis buffer containing 0.05 M Tris-HCl buffer (pH 7.5), 0.15 M NaCl, 0.001 M EDTA, 0.5% NP40, and one cOmplete™ Protease Inhibitor Cocktail Tablet (Roche, Basel, Switzerland) per 10 ml was added to bacterial pellets and incubated for 30 min on ice. The suspension was homogenized using needle sonication and the cell debris and membranes were removed by ultracentrifugation. His-tagged recombinant colZ was purified using immobilized metal ion affinity chromatography (IMAC) with a Ni Sepharose 6 Fast Flow column (GE Healthcare, Chicago, IL, USA) and eluted using an imidazole gradient (0.01–0.3 M) in 0.05 M Tris-HCl buffer (pH 7.5) containing 0.3 M NaCl. Fractions containing colZ were concentrated and transferred to 50 mM phosphate buffer (pH 7.0) using 3 kDa cut-off membrane ultrafiltration, and the protein concentration was determined using a RC DC Protein Assay (Biorad, Hercules, CA, USA).

The activity of the purified colicin Z protein was tested by spotting 10-fold dilutions on agar plates containing a susceptible *E*. *coli* O164. Plates were then incubated at 37 °C overnight and a reciprocal value of the highest dilution of purified colZ causing both clear (complete growth inhibition of the indicator strain) and turbid zones (any detectable growth inhibition of the indicator strain) was used as a description of colicin activity (in arbitrary units, A.U.).

### Determination of colicin Z killing mechanism

The pore-forming activity of colicin Z (concentrations up to 340 nM) was tested using conductivity measurements in 1 M KCl, 10 mM HEPES, pH 6 on black lipid membranes composed of DPhPG, *E*. *coli* polar lipid extract (Avanti Polar Lipids, Alabaster, AL, USA), or soybean lipids (Asolectin-Type II, Merck KGaA, Darmstadt, Germany). Experiments were performed as described previously^50^. The effect of colZ on peptidoglycan was tested using the dye release method described in Zhou *et al*.^51^. Briefly, RBB^3^ (Sigma-Aldrich) dyed purified peptidoglycan (50–300 mg/ml in 0.2 M glycine-NaOH, pH 10.0) isolated from EIEC O164 strain were used for measurement of colicin Z activity. Peptidoglycan (PG) isolation from EIEC O164 was performed according to the protocol described by Benešík *et al*.^52^. The RBB-released dye was determined spectrophotometrically at 595 nm in three independent measurements (each in two technical replicates). The active colicin Z (0.1 mg/ml) and control reactions were incubated with RBB-PG at 37 °C for 24 h. The controls comprised denatured colicin Z (0.1 mg/ml; heat inactivated for 10 min at 100 °C), distilled water, and lysozyme (0.1 mg/ml) (Sigma-Aldrich). In addition, to assess potential contaminants present in the purified colicin Z, *E*. *coli* TOP10F′ containing a control construct represented by an empty pBad/HisB cloning vector was purified in the same way as was the *E*. *coli* TOP10F′ colicin Z producer. The resulting control, absent the colZ, was used at the same protein concentration (0.1 mg/ml).

In addition, the detection of peptidoglycan cleavage products was performed using mass spectrometry. Fractions of control commercial PG, isolated from *E*. *coli* K12 (InvivoGen, Toulouse, France) (1 mg/ml), and PG incubated (37 °C) with denaturated (0.01 mg/ml) or active colicin Z (0.01 mg/ml) in three different time-points (1 min, 5 min, and 1 hour) were mixed with MALDI matrix (12.5 mg.ml^−1^ ferulic acid in water:acetonitrile:formic acid mixture, 50:33:17, v/v), applied to a stainless steel sampling target, and analyzed using MALDI-TOF MS using an Ultraflextreme instrument (Bruker Daltonics, Bremen, Germany) operated in the linear positive ion detection mode.

### Sequence analysis and construction of phylogenetic trees

The DNASTAR Lasergene package was used for sequence analyses (DNASTAR). The Universal Protein Resource (UniProt) database was used for homology searches of proteins encoded on pColZ (The UniProt Consortium, 2017). ORF finder tool (NCBI) was used for prediction of open reading frames on the plasmid ColZ. Colicin gene or amino acid alignments and phylogenetic trees were computed with Molecular Evolutionary Genetics Analysis (MEGA) version 7.0^53^. DoriC 5.0, a database of *ori*C regions was used for identification of the pColZ *ori* site^54^. The I-TASSER server was used for protein structure prediction and structure-based function annotation^55^. For prediction of protein subcellular localization, signal peptide sequence identifications, and prediction of transmembrane segments, ExPASy programs (PSORTb, SignalP-5.0, and TMPred) were used^56^.

## Supplementary information

Table S1

Table S2

Supplementary information Fig. S1, S2, S3, Table S3

## Data Availability

All relevant data are within the Manuscript and its Supporting Information files.
