# Supplementary material for: Colicin Z, a structurally and functionally novel colicin type that selectively kills enteroinvasive Escherichia coli and Shigella strains
Source: Sci Rep. 2019 Jul 31;9:11127. doi: 10.1038/s41598-019-47488-8 (PMC6668396; doi:10.1038/s41598-019-47488-8)
Supplement: Supplementary file 3 — Supplementary information Fig. S1, S2, S3, Table S3 [file 41598_2019_47488_MOESM3_ESM.pdf]

Supplementary information

Colicin Z, a structurally and functionally novel colicin type that selectively kills enteroinvasive *Escherichia coli* and *Shigella* strains

Lenka Micenková[1], Juraj Bosák[2], Jiri Kucera[3], Matěj Hrala[2], Tereza Dolejšová[4], Ondrej Šedo[5], Dirk Linke[6], Radovan Fišer[4], David Šmajs[2*]

[1]Research Centre for Toxic Compounds in the Environment, Faculty of Science, Masaryk University, Kamenice 5, 625 00 Brno, Czech Republic

[2]Department of Biology, Faculty of Medicine, Masaryk University, Kamenice 5, Building A6, 625 00 Brno, Czech Republic

[3]Department of Biochemistry, Faculty of Science, Masaryk University, Kamenice 5, Building A5, 625 00 Brno, Czech Republic

[4]Department of Genetics and Microbiology, Faculty of Science, Charles University, Viničná 5, 128 44 Prague 2, Czech Republic

[5]Central European Institute of Technology, Masaryk University, Kamenice 5, 625 00 Brno, Czech Republic

[6]Department of Biosciences, University of Oslo, P.O. Box 1066, Blindern, 0316 Oslo, Norway

*Corresponding author.
E-mail: dsmajs@med.muni.cz

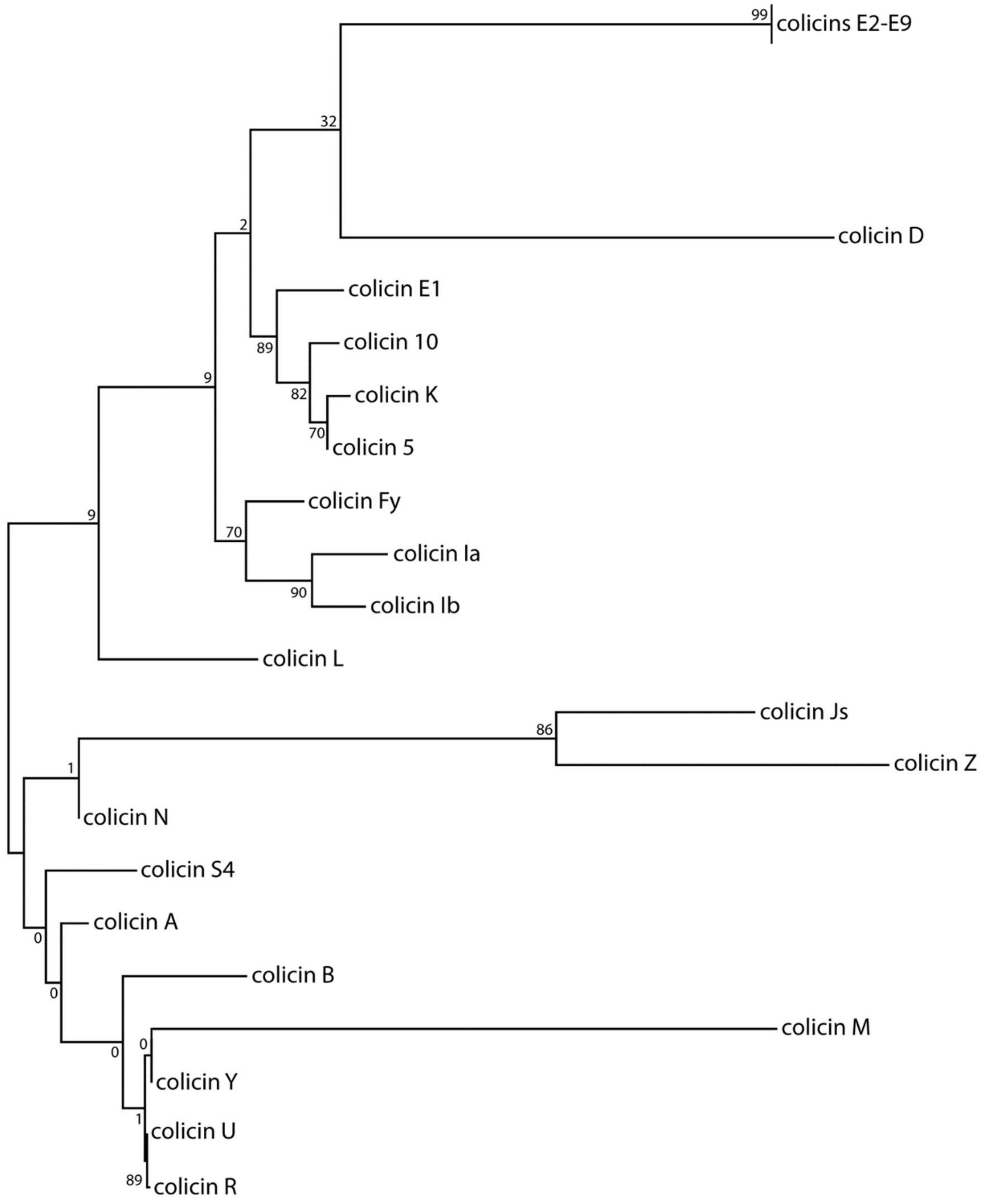

Fig. S1. Phylogenetic tree of amino acid sequences of colicin Z and 25 previously characterized colicin types constructed using the Maximum Likelihood method**.** The bar scale represents 0.5 nucleotide substitutions per site. Bootstrap values based on 1,000 replications are shown next to branches.

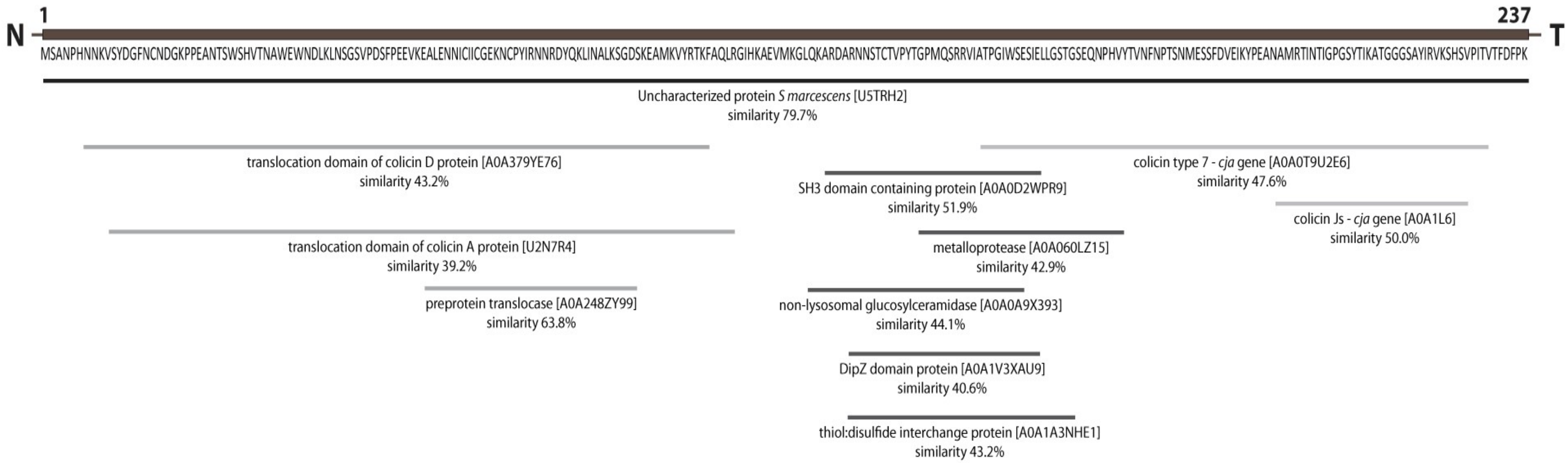

Fig. S2. A detailed domain organization of colicin Z. *predicted translocation, activity, and receptor binding domains **sequence identity (%)

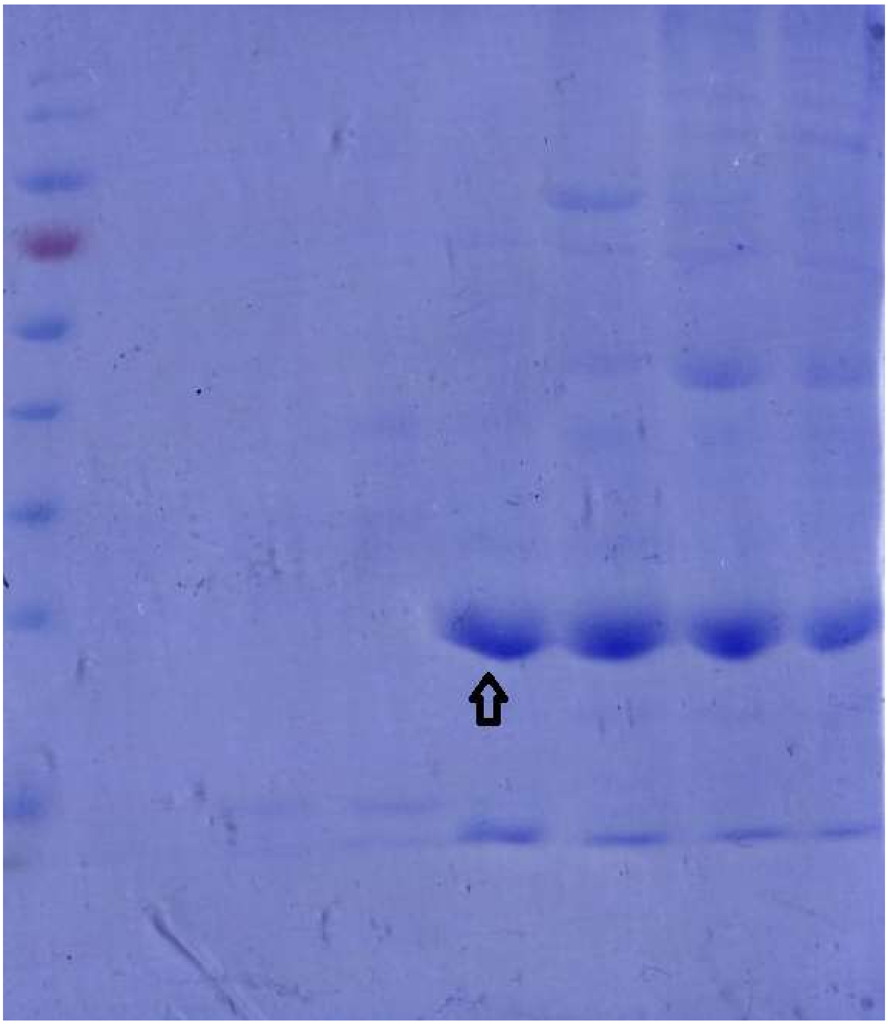

Fig. S3. Purification of colicin Z containing an N-terminal histidine tag by using Ni Sepharose 6 Fast Flow column. Lane 1, low-molecular-weight protein standard (PageRuler Prestained Protein Ladder, Fermentas); lanes 4-7, purified colicin Z with an N-terminal histidine tag: full-length gel.

Table S3. Primers pairs used in this study

Name	Description	Sequence 5'-3'
cza -F	**colicin Z activity gene**	ATGGATCCGAGCTCGAGATCTGCAGATGAGTGCAAACCCGCATA
cza -R		GCTTCGAATTCCCATATGGTACCAGTTACTTAGGAAAATCGAAAGTAA
czi -F	**colicin Z immunity gene**	AGGATCCGAGCTCGAGATCTGCAGATGTTAAATTCGTTCAAGGCGCCG
czi-R		GCTTCGAATTCCCATATGGTACCAGTTAAGAACATTCGATCTTATGCTC
Tn5-F	**colZ - plasmid sequencing**	ACCTACAACAAAGCTCTCATCAACC
Tn5-R		GCAATGTAACATCAGAGATTTTGAG
B1356-1F		TAGTCCAGATTGTCCGCCTG
B1356-1R		CAGGCGGACAATCTGGACTA
B1356-2F		CACCAGCTGGAGCCATGTAA
B1356-2R		TTACATGGCTCCAGCTGGTG
B1356-3F		GCTATGTCGATACTCCCTAC
B1356-3R		GTAGGGAGTATCGACATAGC
B1356-4F		CAGCACGACTCACATGCGTT
B1356-5F		GTGCAGCTTGATTCATGGTG
B1356-5F		GCTCACCATCAACCCGTTTA
B1356-6R		TAAACGGGTTGATGGTGAGC
B1356-7F		CAGTGCTTCCTTCACTTCTT
B1356-7R		AAGAAGTGAAGGAAGCACTG
B1356-8F		CCATAGTGACTGGCGATGCT
B1356-8R		AGCATCGCCAGTCACTATGG
cjrA -F	**colicin Z receptor identification**	ATGGATCCGAGCTCGAGATCTGCAGATGAGAAAGTTCATCCTTATTTCTATGATAACGTT
cjrA -R		GCTTCGAATTCCCATATGGTACCAGTTACCTTTTTGTTGTATCTGGCGCTACAAACCACA
cjrB -F		ATGGATCCGAGCTCGAGATCTGCAGATGATGAATATTCTCCACTTCCCACAGTCGGTAAA
cjrB -R		GCTTCGAATTCCCATATGGTACCAGTTACTCCTTCAACTTAAAGGTAATCGGCATTTTTA
cjrC -F		ATGGATCCGAGCTCGAGATCTGCAGATGAACGTTATAAAACTGGCTATCGGCTCAG
cjrC- R		GCTTCGAATTCCCATATGGTACCAGTTACTCAGAAACGAATGTCCCCGCCAAATATCCAT
